# Improving the efficiency of gasochromic response by using a dual sample system with WO_3_ thin films with Pd and Pt catalysts

**DOI:** 10.1038/s41598-025-31815-3

**Published:** 2025-12-11

**Authors:** Wiktoria Weichbrodt, Des Gibson, Jarosław Domaradzki, Jarosław Serafińczuk, Michał Mazur

**Affiliations:** 1 Faculty of Electronics, Photonics and Microsystems, Wroclaw University of Science and Technology, Janiszewskiego 11/17, 50-372 Wroclaw, Poland; 2 Institute of Thin Films, Sensors and Imaging, University of the West of Scotland, Paisley, PA1 2BE UK

**Keywords:** Chemistry, Energy science and technology, Materials science

## Abstract

Hydrogen, when produced using renewable energy sources, is a zero-emission fuel that does not emit harmful pollutants. Therefore, it is increasingly being researched as an alternative to traditional fossil fuels, although it poses certain risks due to its explosive nature. Gasochromic materials such as tungsten oxide (WO$_{3}$) thin films show promise for passive and remote hydrogen sensing. In this work the gasochromic reaction of WO$_{3}$ thin films with palladium and platinum catalysts, deposited by electron beam evaporation, was investigated using various sample configurations during measurements. Gasochromic measurements revealed that Pd-coated WO$_{3}$ thin films exhibited superior sensor response and faster response and recovery times compared to Pt-coated films, which demonstrated better long-term stability. The aim of this study was to investigate gasochromic properties of a novel configuration consisting of two samples simultaneously mounted on a holder, which enabled a multiplied gasochromic response compared to that of a single sample. Furthermore, this is the first time such an approach has been reported for WO$_{3}$-based systems exhibiting gasochromic properties. Additional experiments confirmed the high selectivity of the sensor toward hydrogen and its excellent long-term stability even after one year. Moreover, in-situ X-ray Diffraction measurements were performed to gain insight into the structural changes occurring during the gasochromic reaction. A universal configuration offering a simple and effective way to significantly enhance gasochromic response was also proposed.

## Introduction

Hydrogen is essential for a long-term strategy to reduce greenhouse gas emissions, both through the deployment of hydrogen technologies and the application of hydrogen in the manufacture of industrial goods^1^. Nonetheless, hydrogen is extremely flammable and explosive (for concentration in the range of 4-75%) and requires very low ignition energy at specific concentrations^2^. Its ability to easily penetrate most materials and diffuse quickly presents significant challenges for containment and storage^3,4^. As a result, reliable, cost-effective, and sensitive systems for detecting hydrogen leaks in real time are essential to ensure safe usage and promote public acceptance^5,6^. Optical sensors stand out from other possible types of hydrogen detection by their passive and intrinsically safe ability to remotely detect hydrogen in inaccessible areas using optical fibers^7–10^.

One of the methods enabling optical detection of hydrogen is the use of gasochromic tungsten oxide films^5,10–13^. WO$_{3}$ gasochromism involves a change in the transparency of the thin film, and the phenomenon usually requires a layer of a catalyst, e.g. palladium, platinum or gold^6,14–18^. In WO$_{3}$ resistive sensors, gas molecules interact with oxygen molecules adsorbed on the surface of the semiconductor, causing a decrease in both carrier concentration and electron mobility, which leads to a change in resistance^19,20^. Resistive and gasochromic sensors represent two different approaches to gas detection. Resistive sensors work by monitoring changes in electrical resistance caused by gas adsorption on the surface of the sensor material, while gasochromic sensors rely on optically detectable colour changes resulting from reversible chemical interaction with target gases. Several potential mechanisms for the gasochromic phenomenon are reported in the literature, including double injections of ions^21–24^. This mechanism is based on spillover effect, hydrogen atoms reaching the surface of the oxide, diffuse into the mass, leading to a reduction of the W$^{6+}$ centre in tungsten oxide lattice to W$^{5+}$, resulting in a blue colour in visible light^23–25^. Furthermore, Georg, Graf, and Wittwer’s gasochromic model is a well-established reference in the field, illustrating the mechanisms of coloring and bleaching processes. Many aspects of this approach appear across related literature^21–23,26,27^. After reaching the WO$_{3}$ surface, Georg suggested that hydrogen atoms enter through pores or grain boundaries, where they create oxygen vacancies and water. The vacancies migrate into the interior, reducing oxygen content and inducing blue coloration. During bleaching, oxygen dissociates at the catalyst surface, diffuses into the WO$_3$structure, fills the vacancies, and the remaining vacancies move back toward the surface^23,26,27^.

Recent studies highlight the significant influence of catalyst type on the performance of WO$_{3}$-based sensing films, particularly in terms of response time, sensitivity, and long-term stability^5,6,13–16,24,25^. Dai et al. in their study successfully improved sensor response employing WO$_{3}$-Pd$_{2}$Pt-Pt composite film, due to the combination of the beneficial features of both catalysts^28,29^. According to the literature, palladium among other catalyst material has shown good selectivity towards hydrogen^30^. In turn, the use of platinum provides enhanced stability^8,31^. Moreover, Yaacoob et al.^25^n their research showed that in the wavelength range from 500 to 800 nm, the use of a palladium catalyst instead of platinum allows to improve sensitivity and accelerate both the colouring and bleaching processes. This topic is certainly more complex, because in this respect the distribution of the catalyst on or in the thin film also plays an important role^8,13,15,32–36^.

In this study, we aimed to enhance the sensing performance of optical hydrogen sensors based on tungsten oxide (WO$_{3}$), a material crucial in climate-neutral economy. This study employed tungsten oxide thin films prepared by electron-beam evaporation and then annealed at $400^\circ$C. After thermal modification of WO$_{3}$ thin film, Pt and Pd catalysts were deposited as adlayers using the same method. The key novelty of this study is the introduction of a two-sample measurement system, i.e. two samples were simultaneously attached to the holder in a cryostat. This configuration allowed to significantly enhanced the gasochromic response by multiplying the effect of both samples exposed to hydrogen. This approach enables an improved sensor response and stability compared to single-sample configurations. The scientific contribution of this work additionally highlights the advantages of both commonly used catalysts. Furthermore, the sensor exhibited high selectivity toward hydrogen and excellent long-term stability, even after one year of repeated cycling. To gain deeper insight into the material behavior, in-situ XRD measurements were performed, revealing structural changes accompanying the gasochromic reaction.

## Experimental details

Using the electron beam evaporation (EBE) method, as detailed in Ref.^37^, tungsten oxide thin films were deposited on unheated substrates of amorphous silica (SiO$_{2}$) and silicon (Si). The distance between the substrate and the source material (WO$_{3}$ pellets with a purity of 99.99 at. % from Kurt J. Lesker) in vacuum chamber was 500 mm. The rotation speed of the substrates was equal to 10 rpm. Oxidation was ensured by additional oxygen flow with a rate of 100 ml/min during the process and the pressure during evaporation was ca. 1.5 $\times$ 10$^{-3}$ Pa. The electron beam voltage and current were set at 6 kV and 20 mA, respectively, resulting in the deposition of thin film with a thickness of ca. 360 nm. After deposition, the WO$_{3}$ films were annealed in a Nabertherm tubular furnace at $400 ^\circ$C for 4 hours in ambient air. The EBE method was also used to deposit a very thin catalyst layer on top of the WO$_{3}$ films. The thickness of catalysts adlayers of 1.5 nm was monitored in situ during the deposition process using a Quartz Crystal Monitor system (Inficon). Finally, two samples of Pd/WO$_{3}$ and Pt/WO$_{3}$ thin films were prepared for further investigations.

The microstructure of the deposited thin films was determined by Raman spectroscopy and X-ray diffraction using a Thermo Fisher Scientific DXRTM Raman microscope equipped with a 455 nm laser diode with a power of 6 mW and a PANalytical Empyrean powder diffractometer with CuK$\alpha _{1}$ source, respectively. The average crystallite size was calculated using the Debye-Scherrer formula with MDI Jade 5.0 software^38^. Using a FEI Helios Xe-PFIB scanning electron microscope, the surface morphology and cross-section of WO$_{3}$ coatings were examined. Nanoscale topography of WO$_{3}$ thin films with and without catalysts was evaluated with a Nanosurf Flex atomic force microscope (AFM) in tapping mode, with analysis done in WSxM 5.0 software^39^. Optical and gasochromic properties were analysed by measurements of the light transmission spectra using an Ocean Optics QE65000 spectrophotometer and a DH-BAL 2000 source with coupled halogen and deuterium lamps in spectral range from 300 to 1000 nm. The samples were placed on a temperature-controlled finger in a cryostat (Janis type) of approximately 1500 cm$^{3}$ capacity. All measurements were carried out at a temperature of $150 ^\circ$C. Hydrogen concentration was precisely controlled using MKS mass flow controllers. Filtered air was injected into the cryostat at rate of 1000 ml/min using an Atlas Copco GX3 F compressor. Measurements were carried out at $150 ^\circ$C and changes in transmission at 900 nm were recorded during exposure to hydrogen in argon at concentrations ranging from 25 to 1000 ppm, while colouring and bleaching cycles lasted 30 minutes. A crucial aspect of the assessment of gasochromic properties is the determination of performance parameters of measured coatings. Two key parameters in this assessment are the change in transmission ($\Delta T$) (1) and the sensor response (*SR*) (2):

1 $$\begin{aligned} \Delta T= & T_{\text {air}} - T_{\textrm{H}_2} \end{aligned}$$

2 $$\begin{aligned} SR= & \frac{T_{\text {air}}}{T_{\textrm{H}_2}} \end{aligned}$$

where $T_{air}$, $T_{\textrm{H}_2}$ are the sample transmission value in air and gas, respectively for a given light wavelength.

The 90% response and recovery times were measured for two selected concentrations of 25 and 100 ppm. The change of the chemical state of the surface, i.e. W4f region, due to the in-situ hydrogen exposure of Pd/WO$_{3}$ and Pt/WO$_{3}$ thin films was evaluated by X-ray photoelectron spectroscopy studies performed with the use of a Specs Phoibos 100 hemispherical analyser equipped with a non-monochromatic MgK$\alpha$ beam. The in-situ hydrogen exposure was done for both samples for 30 minutes at a temperature of $150 ^\circ$C. Detailed scans of the W4f, Pd3d and Pt4f regions, with a resolution of 0.1 eV, were taken before and after in-situ H$_{2}$) exposure. The spectra were calibrated using the adventitious C1s peak (284.8 eV). Analysis was performed using CasaXPS software, version 2.3.24PR1.0. A Shirley background and a Gaussian-Lorentzian peak shape (70:30) were applied to analyse the W4f region, while a Tougaard background was used for the Pd3d and Pt4f regions. In-situ grazing-incidence XRD measurements were performed with WO$_{3}$ thin films deposited on amorphous SiO$_{2}$ using a Malvern Panalytical Empyrean diffractometer (Cu K$\alpha _1$, $\lambda =1.5406$Å) with a PIXcel 3D detector. Samples were mounted in an Anton Paar TTK-600 chamber for controlled gas flow (1000 ppm H$_{2}$ in Ar) at $150 ^\circ$C. Data were collected at a fixed incidence angle of $3^\circ$, with a $0.28^\circ$ parallel-plate collimator on the diffracted beam. Sequential scans were acquired in air (during heating to $150 ^\circ$C) and under H$_{2}$ over $2\theta$ = 20–$60^\circ$, step $0.2^\circ$, 3 s per step.

## Results

**Figure 1:**
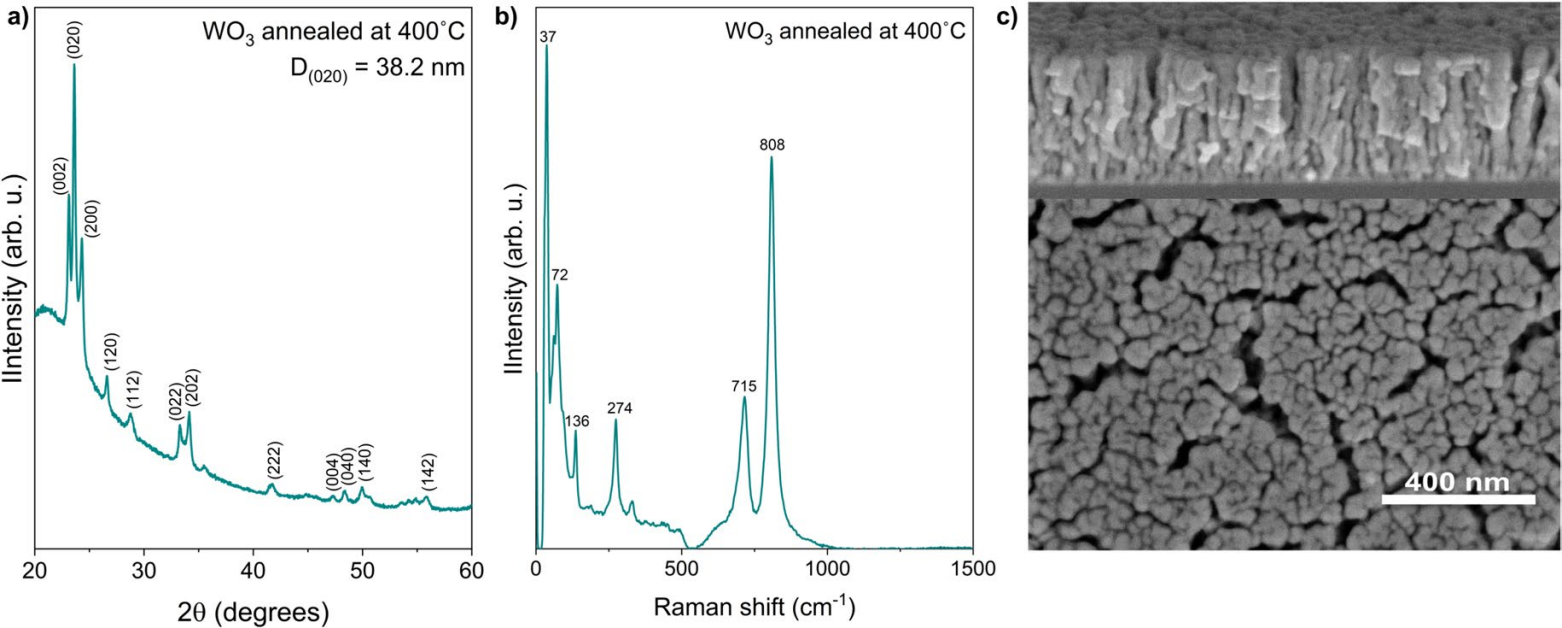
(**a**) XRD pattern, (**b**) Raman spectrum, and (**c**) SEM images of tungsten oxide thin film annealed at $400 ^\circ$C.

The thin films of tungsten oxide after annealing were characterized by means of X-ray diffraction, and the resulted diffractogram is shown in Fig. 1a. The XRD pattern of thin film is assigned to monoclinic WO$_{3}$ phase evidenced by the high intensity and sharp peaks at $2\theta$ of $23.1^\circ$, $23.6^\circ$ and $24.3^\circ$ that correspond to (002), (020) and (200) planes. Crystallites size was lower than 40 nm. Figure 1b present the results of Raman analysis. Raman spectrum shows peaks of high intensity in two different regions. Peaks observed at lower Raman shifts of 37, 72, 136 cm$^{-1}$ are attributed to lattice vibrations, at 274 cm$^{-1}$ to bending vibrations and at 715, 808 cm$^{-1}$ correspond to the stretching vibration^40,41^. Polycrystalline WO$_{3}$, in both monoclinic and triclinic crystalline phases, typically shows peaks at 270, 718, and 807 cm$^{-1}$^42^, which confirm results obtained by X-ray diffraction. The morphology of WO$_{3}$ film is shown on SEM image in Fig. 1c. The annealed at $400^\circ$C tungsten oxide thin films contained voids between its grains, resulting in a porous structure. In the cross-section, the structure is fibrous, the mean fibre diameter were equal to 52.3 nm with a standard deviation of 10.8 nm. Furthermore, the thickness of 360 nm was determined by cross-section SEM image.

**Figure 2:**
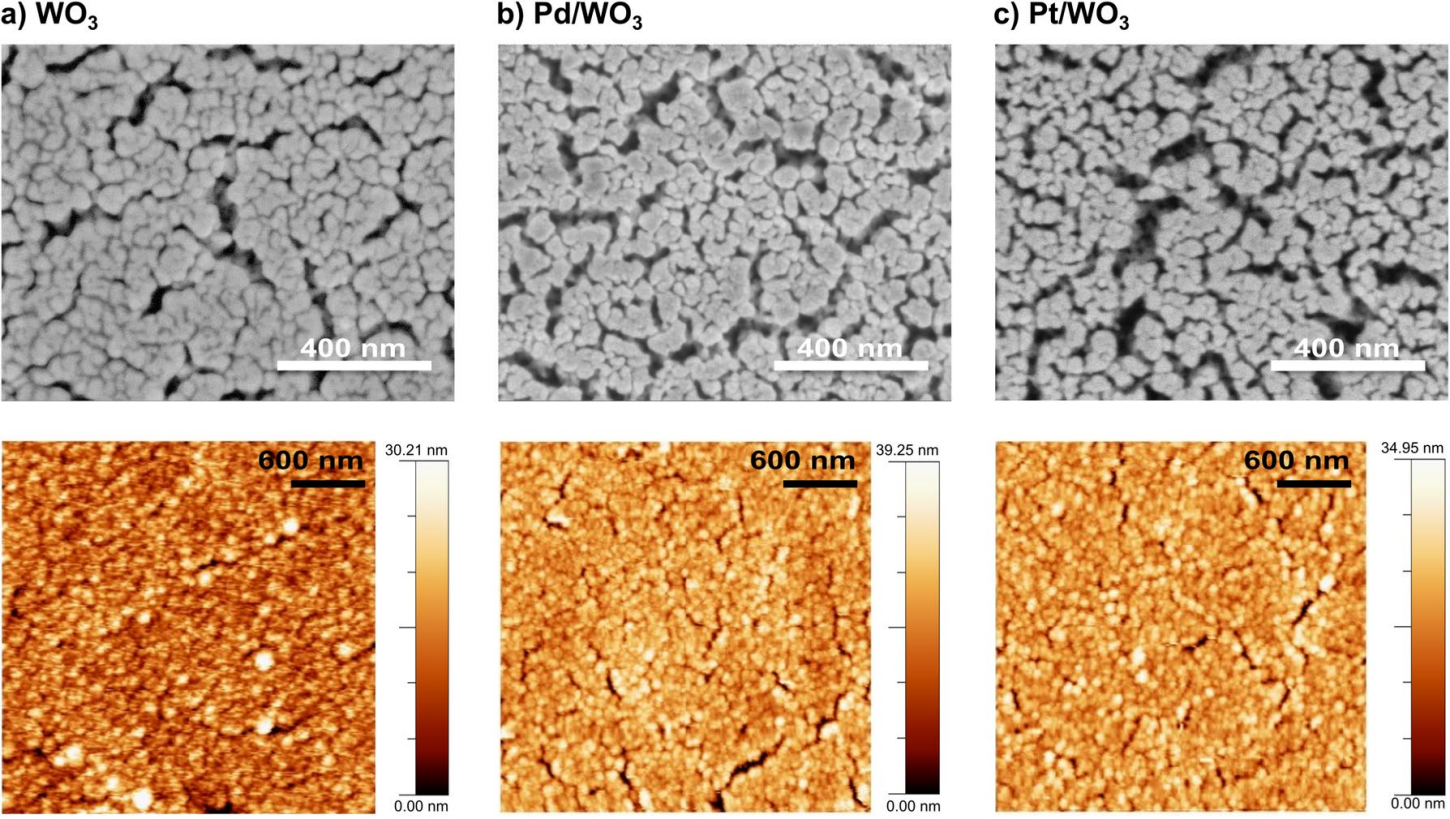
SEM and AFM images of (**a**) WO$_{3}$, (**b**) Pd/WO$_{3}$, (**c**) Pt/WO$_{3}$.

The surface morphology after catalyst deposition was also examined by scanning electron and atomic force microscopy (Fig. 2). The SEM and AFM surface images are very similar for deposited thin films with visible voids between the fibres. Palladium and platinum catalyst adlayers are so thin that they were not observed in SEM images. The deposition of the catalyst caused a slight change, i.e. an increase in the roughness of the samples. The RMS value is slightly above 5 nm and increases for palladium by only 0.1 nm and for platinum by 0.4 nm.The small differences in RMS roughness may be due to measurement uncertainty or sampling different grains/regions. However, the slightly granular texture observed together with the modest increase in RMS are consistent with Volmer–Weber island growth of discontinuous Pd and Pt nanoparticle catalyst adlayers.

**Figure 3:**
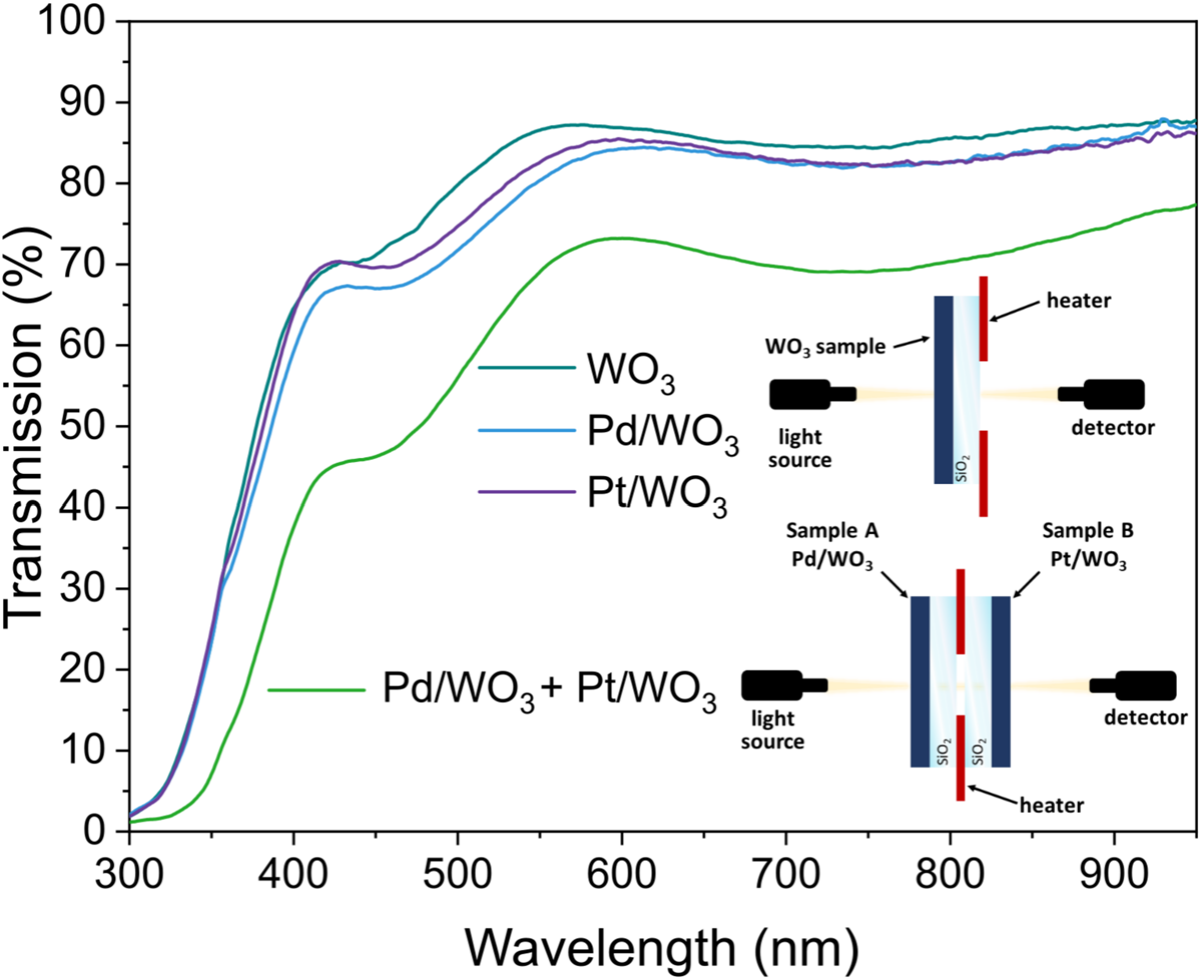
Transmission spectra measurement results of one-sample and two-samples configurations of WO$_{3}$ thin films.

The investigation of the optical properties began with the determination of the transmission spectrum, as shown in Fig. 3 WO$_{3}$ thin film after thermal modification at $400 ^\circ$C is characterised by high transparency in the visible and near infrared regions. Deposition of catalysts to the film resulted in a slight decrease in transmission values at wavelengths above 400 nm, which may suggest a slightly larger deposition volume. Moreover, sample with palladium adlayer had a marginally lower transmission than the sample with platinum in the visible wavelength range. Transmission spectrum was also measured for two-samples configuration and a schematic view of the system is shown in Fig. 3. Due to the use of two sample configurations, the transmission decrease is evident because the final transmittance is actually the result of multiplying the light transmission coefficients of the Pd/WO$_{3}$ and Pt/WO$_{3}$ thin films.

**Figure 4:**
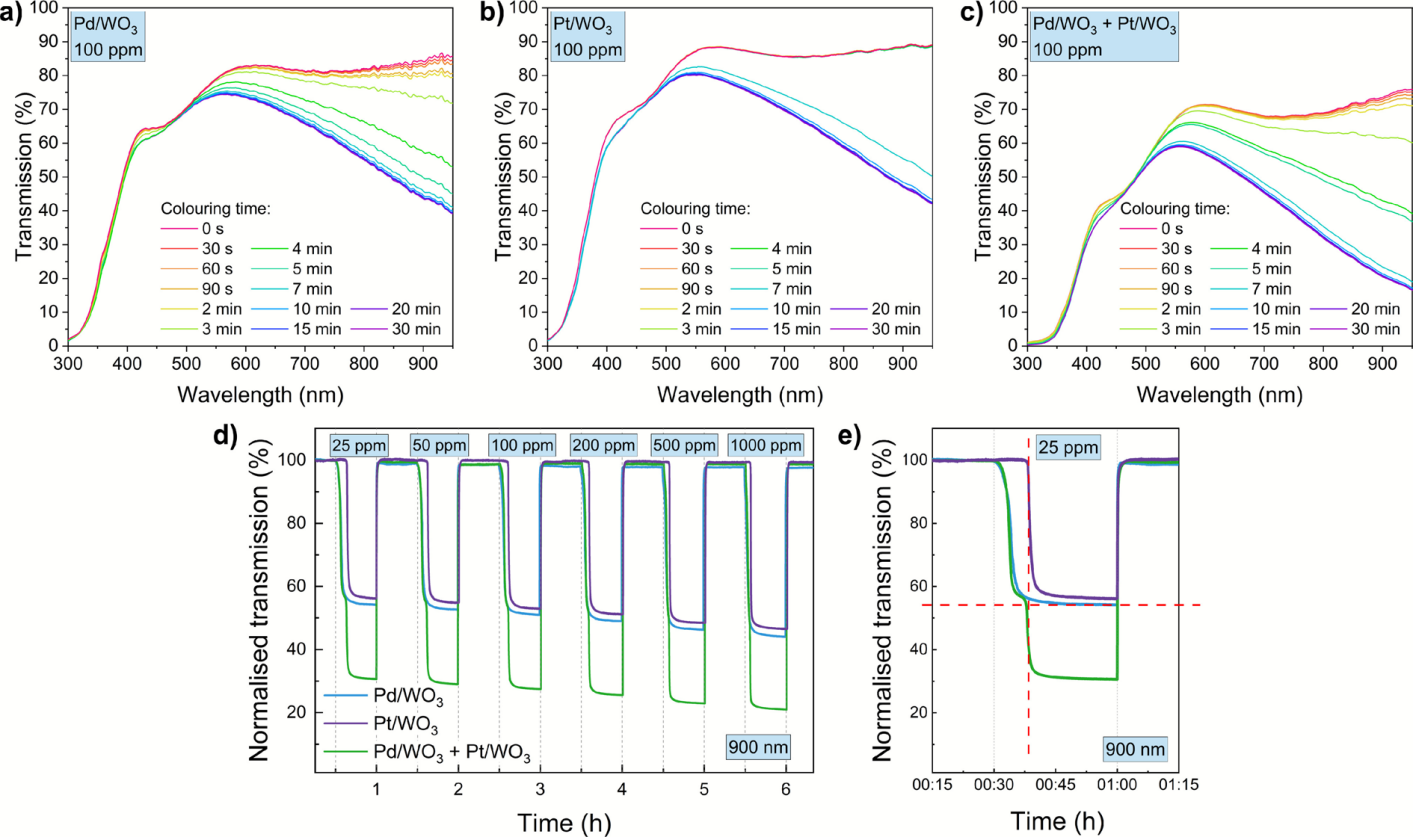
Transmission spectra of WO$_{3}$ thin films of one-sample and two-samples configurations during hydrogen exposure with a concentration of 100 ppm: (**a**) Pd/WO$_{3}$, (**b**) Pt/WO$_{3}$ and (**c**) Pd/WO$_{3}$+Pt/WO$_{3}$, normalised transmission values at 900 nm during hydrogen exposure for different configurations of WO$_{3}$ thin films: (**d**) normalised gasochromic response recorded for selected H$_{2}$ concentrations, (**e**) an exemplary response waveform for 25 ppm H$_{2}$ concentration illustrating the difference in optical response for Pt/WO$_{3}$ and Pd/WO$_{3}$ layers and the Pt/WO$_{3}$ + Pd/WO$_{3}$ system.

A gasochromic effect was observed for all configurations in the wavelength range above 400 nm for the tested hydrogen concentrations. Irrespective of the type of catalyst or configuration used, the difference between the transmission value in the bleached and coloured states above 500 nm also varies with increasing wavelength. For 100 ppm of hydrogen in air, the transmission value at 900 nm of the films with palladium or platinum decreased from more than 85% to less than 50%, while for the two-sample configuration from about 73% to 20% (Fig. 4a–c). Normalised transmission values over time during the colouring and bleaching cycles were measured for hydrogen concentrations of 25, 50, 100, 200, 500 and 1000 ppm for 900 nm (Fig. 4d). Gasochromic performance was good, with efficient colouring dynamics observed. It is worth noting that the gasochromic process for the Pd/WO$_{3}$ film started almost immediately after exposure to hydrogen for the tested concentrations. On the other hand, in the case of the WO$_{3}$ thin film covered with a platinum catalyst, the change in transmission starts only after a few minutes – but the process initiates faster with increasing concentration. The final value of the Pd/WO$_{3}$ transmission in the coloured state is lower than for Pt/WO$_{3}$, however, for the palladium film a decrease in baseline with successive cycles is also evident. Better stability of platinum is consistent with findings in the literature^8^. For the two-samples configuration, an almost instantaneous change in transmission in response to hydrogen was also noted. For 25 ppm hydrogen, after about 5 minutes the response starts to become stable, then after about 7 minutes a rapid decrease in transmission is noticeable again (Fig. 4e). This behaviour is directly related to the rate of gasochromic process of samples with different catalysts. First, the response from the Pd/WO$_{3}$ is observed and then Pt/WO$_{3}$ starts to respond to hydrogen. As the hydrogen concentration increases, the gasochromic process occurs faster, so that in subsequent cycles for the two-samples system, the step in the transmission line is no longer observed. For the two-samples configuration, a decrease in baseline over time was also noticed, however less than for Pd/WO$_{3}$.

Table 1 summarizes the results of the determined gasochromic performance. The gasochromic properties was calculated using Eqs. (1), (2). Pd/WO$_{3}$ film showed better performance compared to the Pt/WO$_{3}$ film, especially considering the response time. When two coated glass substrates are mounted facing each other in the cryostat, each acts as an independent optical element. Because both samples are approximately half millimeter thick and slightly tilted, the illumination is broadband (300-1000 nm) and fiber-coupled, the optical phase between the two plates becomes randomized. Under typical conditions of incoherent coupling (i.e. coherent within each thin film, but incoherent between the two substrates), the total transmitted intensity can be approximated by multiplying the individual transmittances together (Eq. 3):

3 $$\begin{aligned} SR_{total} = SR_{\mathrm {Pd/WO_3}}(\lambda ) \cdot SR_{\mathrm {Pt/WO_3}}(\lambda ) \end{aligned}$$

where $SR_{\mathrm {Pd/WO_{3}}}(\lambda )$ and $SR_{\mathrm {Pt/WO_{3}}}(\lambda )$ are the $SR_{\textrm{total}}$ values for thin films with proper catalyst adlayers. Based on theoretical calculation (Eq. 3) for the two-sample configuration, the SR for 25 ppm should be 3.28 and for 100 ppm 3.65. The difference between the theoretical and measured values is minor and probably due to the measuring set-up. Furthermore, the response and recovery times for the two-sample configuration were shorter than for the Pt/WO$_{3}$ film itself.

**Table 1:** Performance parameters for gasochromic WO$_{3}$ thin films for a wavelength of 900 nm.

	*SR*		$\Delta T$		Response time		Recovery time	
	(-)		(%)		(s)		(s)	
H$_{2}$ concentration (ppm)	25	100	25	100	25	100	25	100
Pd/WO$_{3}$	1.84	1.93	38.9	42.0	358	334	8	8
Pt/WO$_{3}$	1.78	1.89	37.9	41.5	609	478	27	43
Pd/WO$_{3}$ + Pt/WO$_{3}$	3.26	3.64	51.5	53.1	573	433	23	35

The correction from multiple reflections between the glass–air interfaces is negligible because of the low Fresnel reflectance and finite detector aperture. As the sensor response is defined as the ratio of transmission in air to transmission in hydrogen, the same multiplicative rule applies (Eq. 4):

4 $$\begin{aligned} SR_{total} = \frac{T^{air}_{Pd/WO_{3}}}{T^{H_{2}}_{Pd/WO_{3}}} \cdot \frac{T^{air}_{Pt/WO_{3}}}{T^{H_{2}}_{Pt/WO_{3}}} = SR_{\text {Pd/WO}_{3}}(\lambda ) \cdot SR_{\mathrm {Pt/WO}_{3}}(\lambda ) \end{aligned}$$

where *T* are transmission values for thin films with adlayers of suitable catalysts in air or hydrogen. Using the measured single-sample responses from Table 1 (Pd/WO$_{3}$ and Pt/WO$_{3}$ at 900 nm), the predicted and measured total responses of the dual-sample system agree within <1% (Table 2).

**Table 2:** Comparison of the measured and theoretically calculated total SR values with relative error.

H$_{2}$ concentration (ppm)	SR$_{Pd/WO_{3}}$	SR$_{Pt/WO_{3}}$	$SR_{\text {Pd}}(\lambda ) \cdot SR_{\textrm{Pt}}(\lambda )$ - theoretical	$SR_{\text {Pd}}(\lambda ) \cdot SR_{\textrm{Pt}}(\lambda )$ - measured	Relative error
25	1.84	1.78	3.28	3.26	− 0.6%
1000	1.93	1.89	3.65	3.64	− 0.3%

According to theoretical assumptions, using a two-sample configuration resulted in better gasochromic properties compared to a single-sample configuration. By using WO$_{3}$ thin film with palladium on one side of the holder, a quicker initiation of the gasochromic effect was achieved, while by using a thin film with platinum on the other side of the holder in a cryostat, improved stability of the sensor over time was ensured.

**Figure 5:**
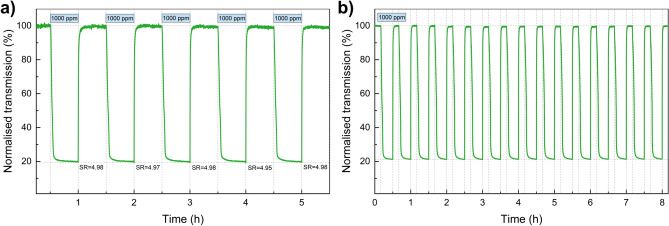
Normalized transmission at 900 nm during exposure to 1000 ppm H$_{2}$ for the Pd/WO$_{3}$ + Pt/WO$_{3}$ dual-sample configuration: (**a**) five consecutive cycles (short-term repeatability); (**b**) 16 cycles recorded one year later on the same sensor (long-term stability).

Figure 5a shows the cycling performance for five consecutive cycles with exposure to 1000 ppm of hydrogen of the two sample configuration. The deviation from the arithmetic mean of the calculated sensor response was a maximum of 2%. The results show consistent sensor performance across all cycles, indicating good repeatability. The long-term stability of the WO$_{3}$ dual-sample configuration was evaluated one year after the initial measurements. A total of 16 gas exposure cycles were performed, each consisting of 20 minutes of colouration in H$_{2}$ and 10 minutes of bleaching in air (Fig. 5b). The final optical transmission decreased to approximately 21.3%, which is slightly above the level of the initial measurement, but this difference can be attributed to the shorter exposure time (by approximately 10 minutes). The baseline at the end of each cycle, i.e. final transmission values between successive cycles, differed by no more than 0.1%. This confirms that the dual-sample WO$_{3}$ system exhibits excellent cycling stability and minimal drift over extended operation time.

**Figure 6:**
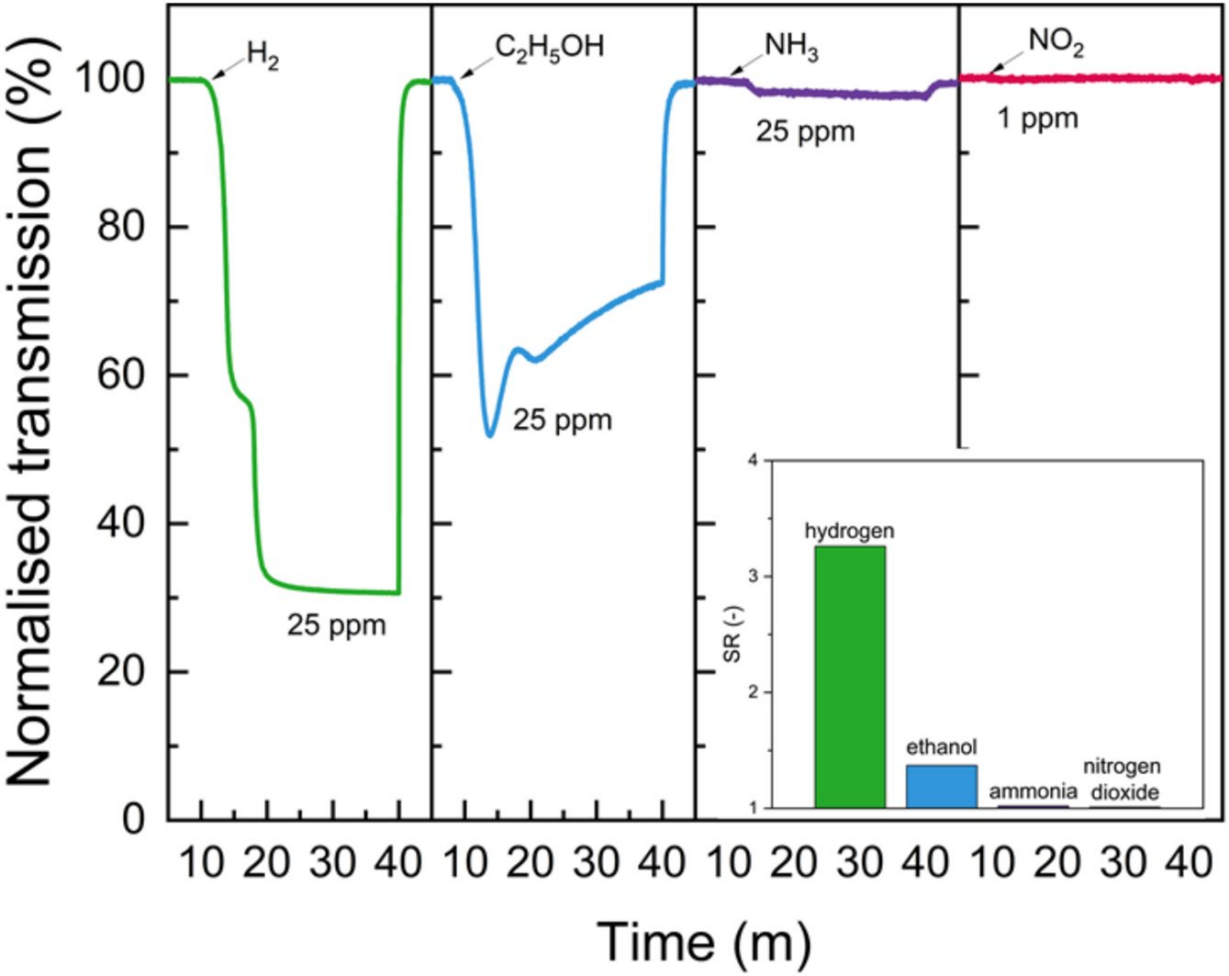
Gasochromic selectivity of the Pd/WO$_{3}$ + Pt/WO$_{3}$ dual sample sensors at 900 nm toward different gases. Inset: sensor response comparison, confirming the dominant selectivity toward H$_{2}$.

Selectivity measurements of Pd/WO$_{3}$ + Pt/WO$_{3}$ dual system were performed, and the results are presented in Fig. 6. The plot shows the normalized optical transmission of the film during exposure to 25 ppm of H$_{2}$, C$_{2}$H$_{5}$OH, NH$_{3}$, and 1 ppm of NO$_{2}$. A pronounced decrease in transmission is observed only in the presence of hydrogen, indicating a strong gasochromic response and high selectivity towards H$_{2}$. In contrast, exposure to ethanol, ammonia, and nitrogen dioxide causes unstable or minor optical changes or none at all. The bar chart compares the sensor response (SR) values, confirming that hydrogen causes the largest and most stable change in optical transmission.

**Table 3:** Comparison of gasochromic properties of tungsten oxides with previous research in the literature.

Sample	Morphology	Method	Carrier gas	Hydrogen concentration	Wavelength	Gasochromic performance	Ref.
Pd/WO$_{3}$ + Pt/WO$_{3}$	Columnar	Electron beam evaporation	Ar	0.01%	900 nm	$\Delta R = 72.2\%$ $t_1 = 433\ \textrm{s}$ $t_2 = 35\ \textrm{s}$	This work
Pt/WO$_{3}$	Nanowires	Hydrothermal method	Ar	1%	750 nm	$\Delta T = 86.2\%$	^43^
Pt/WO$_{3}$	Dense	Low-temperature chemical fabrication	Ar	4%	670 nm	$\Delta T = 70\%$ $t_1 = 5\ \textrm{s}$ $t_2 = 5\ \textrm{s}$	^44^
Pt/WO$_{3}$	Mesoporous	Sulfuric acid post treatment	Ar	4%	1000 nm	$\Delta T = 53.8\%$ $t_1 = 2.4\ \textrm{s}$ $t_2 = 110\ \textrm{s}$	^45^
Pt/WO$_{3}$	Porous	Sol-gel	Ar	4%	1500 nm	$\Delta T = 28.6\%$ $t_1 = 7\ \textrm{min }$ $t_2 = 18\ \textrm{min}$	^46^
Pt/WO$_{3}$	Porous	Colloidal template method	Ar	4%	1500 nm	$\Delta T = 28.6\%$ $t_1 = 66.3\ \textrm{s}$	^47^
Pt/WO$_{3}$	Hierarchical	Solvothermal method	Ar	4%	1500 nm	$\Delta T = 65.1\%$ $t_1 = 24.8\ \textrm{s}$	^47^
Pt/WO$_{3}$	Nanowires	Solvothermal method	Ar	4%	1000 nm	$\Delta T = 76.2\%$ $t_1 = 15\ \textrm{s}$ $t_2 = 53.1\ \textrm{s}$	^48^
Pd/WO$_{3}$	Nanorods	GAD method	Ar	5%	800 nm	$\Delta T = 24\%$ $t_1 = 14\ \textrm{s}$ $t_2 = 1\ \textrm{s}$	^5^
Pt/WO$_{3}$	Nanoporous	Sol-gel	Ar	10%	1000 nm	$\Delta T ~ 30\%$ $t_1 = 20\ \textrm{s}$ $t_2 = 10\ \textrm{s}$	^49^
Pt/WO$_{3}$	Nanorods	Hydrothermal method	Ar	10%	750 nm	$\Delta T = 78.47\%$ $t_1 = 3\ \textrm{s}$ $t_2 = 600\ \textrm{s}$	^50^
Designations: $\Delta T$ - change of transmittance, $\Delta R$ - change of reflectance, $t_1$ – response time, $t_2$ – recovery time							

The obtained results of the gasochromic properties of the tungsten oxide films were compared with the literature (Table 3). The gasochromic properties of the tungsten oxide films were compared with each other, based on parameters such as: $T_{1}$ - response time; $T_{2}$ - recovery time; $\Delta T$ - difference between the transmission value of the films in the coloured and bleached states. Above all, the response times obtained in the present work are significantly larger than those obtained by others^5,44,45,47–50^. It is worth noting that the response of the films to hydrogen at a concentration of 1% or 4% in argon^5,43–50^ is mostly measured in the literature, which is related to its explosive limit. In the present study, the response of WO$_{3}$ thin films to H$_{2}$ concentrations ranging from 0.0025% to 0.1% was investigated. These values are more than a few hundred times lower. During the tests, a decrease in both response and recovery times and an increasing difference between film transmission values with an increase in hydrogen concentration in the measurement atmosphere were observed. This indicates that faster response times would also be achieved at higher hydrogen concentrations. Moreover, by using two sample system, the same or greater change was obtained for a concentration of 0.01% than others for 4%, 5% and 10% hydrogen in argon^5,44–47,49,50^.

**Figure 7:**
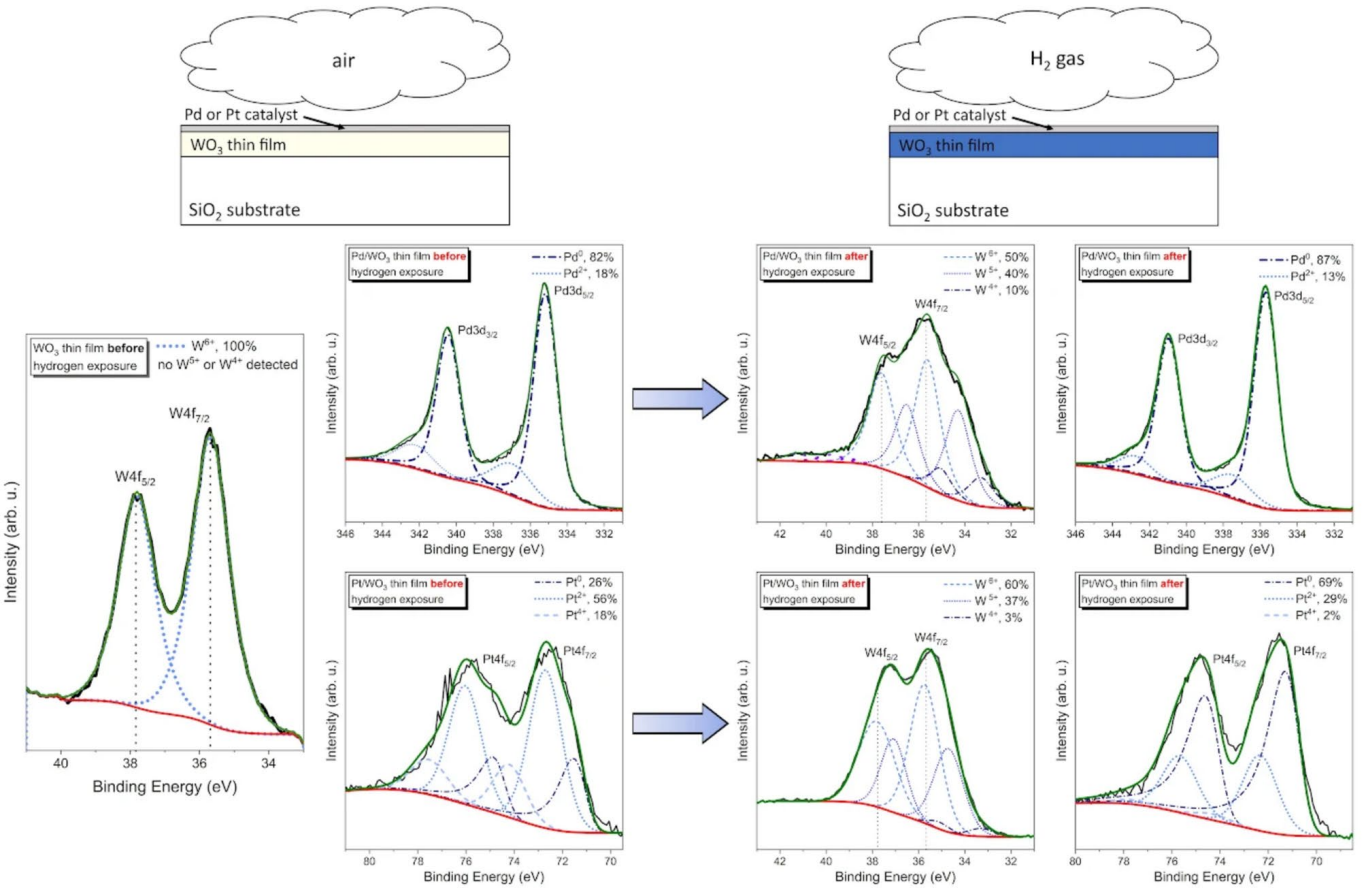
Changes of the tungsten oxidation state measured with the XPS before and after in-situ hydrogenation process.

The change of the oxidation state of tungsten on the surface of Pd/WO$_{3}$ and Pt/WO$_{3}$ thin films was measured by in-situ X-ray photoelectron spectroscopy. It was found that both as-deposited thin films consisted of tungsten only in the 6+ oxidation state as the binding energy of W4f$_{7/2}$ was equal to 35.7 eV (Fig. 7). Additionally, the W4f region showed a spin-orbit doublet of W4f$_{7/2}$ and W4f$_{5/2}$ with a binding energy difference of $\Delta$BE=2.1 eV. This doublet was deconvoluted by two peaks with FWHM in the range of 1.24-1.28 eV and an area ratio of W4f$_{7/2}$ to W4f$_{5/2}$ equal to 1.33, which is in a good agreement with literature and confirms stoichiometry of the as-deposited WO$_{3}$ thin films^51–53^. After the hydrogenation process, a reduction of W$^{6+}$ to W$^{5+}$ and W$^{4+}$ was observed (Fig. 7). The W4f$_{7/2}$ peak was deconvoluted into three components at ca. 35.7 eV, 34.5 eV and 33.3 eV attributed to W$^{6+}$, W$^{5+}$ and W$^{4+}$ ions, respectively^54^. Moreover, in the case of W4f$_{5/2}$ peak, three components at ca. 37.6 eV, 36.6 eV and 35.1 eV were also attributed to W$^{6+}$, W$^{5+}$ and W$^{4+}$ ions, respectively. More pronounced reduction was found for Pd/WO$_{3}$ than for Pt/WO$_{3}$, i.e. the content of W$^{5+}$ and W$^{4+}$ ions was much higher. After a proper deconvolution, the relative components areas for W$^{6+}$, W$^{5+}$ and W$^{4+}$ were determined to be ca. 50%, 40% and 10% for Pd/WO$_{3}$, compared to 60%, 37% and 3% for Pt/WO$_{3}$. These results are consistent with gasochromic studies, where a better gasochromic response and shorter response and recovery times were obtained for Pd/WO$_{3}$ thin films, which can also be attributed to a much more significant change in the oxidation state of tungsten compared to Pt/WO$_{3}$ samples. Before the exposure to hydrogen the Pd catalyst was mostly in the form of metallic Pd$^{0}$ with a most distinctive peak at ca. 335.2 eV in the Pd3d region, while the shoulder peak related to Pd$^{2+}$ was also visible at 336.9 eV^55,56^. The relative content of Pd$^{0}$ and Pd$^{2+}$ was estimated to 82% and 18%, respectively. After exposure to hydrogen the peaks were deconvoluted in the same manner, while their relative content was 87% and 13%, respectively testifying about the reduction of Pd to more metallic one. In the case of Pt catalyst before the exposure to hydrogen, the Pt4f region was deconvoluted into peaks related to the doublets of Pt$^{0}$ ( 71.0 eV), Pt$^{2+}$ ( 72.4 eV) and Pt$^{4+}$ ( 74.3 eV)^55,57–59^ and their relative content was equal to 26%, 56% and 18%, respectively. Upon exposure to hydrogen the composition changed significantly to the more metallic nature, where the relative content of Pt$^{0}$, Pt$^{2+}$ and Pt$^{4+}$ was 69%, 29% and 2%, respectively.

**Figure 8:**
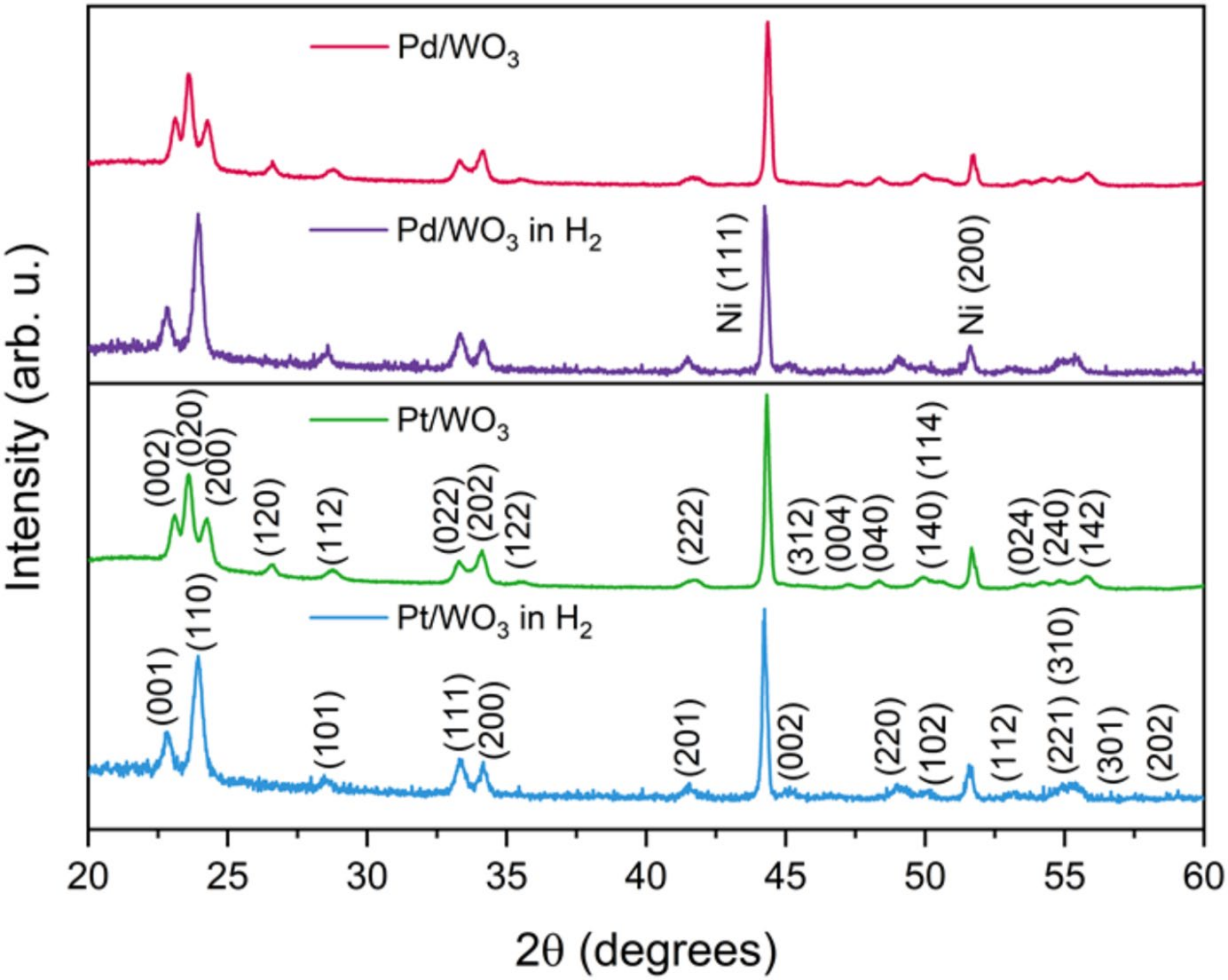
Results of XRD measurements of the WO$_{3}$ structures obtained under ambient conditions and at $150 ^\circ$C in a hydrogen-containing atmosphere.

To gain further insight into the structural changes that accompany the gasochromic effect, in-situ grazing-incidence XRD measurements were performed in a hydrogen-containing atmosphere (1000 ppm $\hbox {H}_{2}$ in Ar at $150 ^\circ$C). As illustrated in Fig. 8, reversible structural transformations were observed in both the $\hbox {Pd/WO}_{3}$ and $\hbox {Pt/WO}_{3}$ films during hydrogen exposure. The initial diffraction pattern of monoclinic $\hbox {WO}_{3}$ ($\gamma$-$\hbox {WO}_{3}$, $\hbox {P2}_{1}$/n), characterised by reflections near $2\theta \approx 23.1^\circ$, $23.6^\circ$ and $24.3^\circ$, evolved into a tetragonal $\hbox {H}_{x}\hbox {WO}_{3}$-type phase with peaks shifted to lower angles ($2\theta \approx 22.7^\circ$, $24.0^\circ$), indicating lattice expansion due to hydrogen diffusion. The Ni(111) and Ni(200) reflections originate from the nickel sample holder inside the TTK-600 chamber. These results demonstrate that the incorporation of hydrogen leads to a transient structural change consistent with the formation of $\hbox {H}_{x}\hbox {WO}_{3}$ (tungsten bronze), providing direct evidence that the observed optical changes (darkening) arises from hydrogen-induced lattice expansion and the partial reduction of $\hbox {W}^{6+}$ to $\hbox {W}^{5+}$/$\hbox {W}^{4+}$. The identical structural response of the samples containing Pd and Pt catalysts confirms that the kinetics are primarily determined by the catalyst, while the gasochromic mechanism of $\hbox {WO}_{3}$ remains unchanged for both types of catalyst. These crystallographic modifications reflect the incorporation of hydrogen atoms into the WO$_{3}$ lattice and the accompanying reduction of tungsten ions from W$^{6+}$ to W$^{5+}$, which is directly confirmed by the XPS spectra.

## Conclusion

In this work tungsten oxide thin films fabricated by EBE method were successfully used as gasochromic coatings. Thin adlayers of platinum and palladium were used as catalysts. Structural studies conducted by X-ray diffraction and Raman spectroscopy confirmed the nanocrystalline structure of the films, and the fibrous morphology of the coatings was determined by cross-section SEM images. AFM analysis and SEM images allowed to conclude that the 1.5 nm thick catalyst does not significantly affect the morphology or roughness of the films. The palladium-coated WO$_{3}$ film exhibited better gasochromic properties for the concentrations tested in terms of sensitivity, response time and recovery time, which was also stated by the in-situ XPS studies. In contrast, the WO$_{3}$ film with applied platinum showed greater stability over time. Additional experiments confirmed the sensor’s high selectivity towards hydrogen, while its response to other gases remained insignificant. The dual system also maintained stable performance after one year. Furthermore, in-situ XRD analyses revealed structural changes occurring in WO$_{3}$ during exposure to gas, confirming the reversibility of the gasochromic process. The use of a two-sample configuration enabled the combination of the beneficial properties of the catalysts, providing a sensor with improved sensor response and stability. This configuration is universal, allowing the use of films with different morphologies, crystallinity, depending on the application.

In future work, it will be particularly interesting to examine the gasochromic properties of WO$_{3}$ thin films of different thicknesses or deposited by other techniques, e.g. magnetron sputtering. This would provide a better understanding of how the deposition process influences the structure, morphology, and gasochromic behavior of the tungsten oxide.

## Data Availability

The data presented in this study are available on request from the corresponding author.
